# Evaluation of the efficacy of manual soft tissue therapy and therapeutic exercises in patients with pain and limited mobility TMJ: a randomized control trial (RCT)

**DOI:** 10.1186/s13005-023-00385-y

**Published:** 2023-09-08

**Authors:** Magdalena Gębska, Bartosz Dalewski, Łukasz Pałka, Łukasz Kołodziej

**Affiliations:** 1 Department of Rehabilitation Musculoskeletal System, Pomeranian Medical University, Szczecin, 70-204 Poland; 2 Department of Dental Prosthetics, Pomeranian Medical University, Szczecin, 70-204 Poland; 3 Private Dental Practice, Zary, 68-200 Poland

**Keywords:** Physiotherapy, Manual therapy, Massage, Surface electromyography, Therapeutic exercises, Temporomandibular joint, Orofacial pain, TMD, sEMG

## Abstract

**Summary:**

The limited number of randomized controlled trials (RCTs) comparing the efficacy of soft tissue manual therapy and self-therapy interventions prompted the authors to focus on the analgesic and myorelaxant use of massage, post-isometric muscle relaxation (PIR) and therapeutic exercise in TMD patients.

**Objectives:**

To evaluate the effectiveness of soft tissue therapy and therapeutic exercises in female patients with pain, increased masseter muscle tension and limited mandibular mobility.

**Material and methods:**

The study was conducted on a group of 82 women (G1) with the Ib disorder diagnosed in DC/TMD (Ib—myofascial pain with restricted mobility). The control group (G2) consisted of 104 women without diagnosed TMDs (normal reference values for TMJ ROM and masseter muscle sEMG bioelectric activity). Diagnostic procedures were performed in both groups (sEMG of the masseter muscles at baseline and during exercise, measurement of TMJ mobility, assessment of pain intensity—NRS scale). The G1 group was randomly divided into 3 therapeutic groups in which the therapy was carried out for 10 days: therapeutic exercises (TE), manual therapy – massage and therapeutic exercises (MTM_TE), manual therapy – PIR and therapeutic exercises (MTPIR_TE). Each time after therapy, the intensity of pain and TMJ mobility were assessed. Sealed, opaque envelopes were used for randomization. After 5 and 10 days of therapy, bilateral sEMG signals of the masseter muscles were acquired.

**Results:**

Massage, PIR and self-therapy led to a decrease in sEMG at rest as well as in exercise. After day 6 of therapy, the groups obtained a significant difference (*p* = 0.0001). Each of the proposed forms of therapy showed a minimal clinically significant difference (MID) in the sEMG parameter at the endpoint, with the most considerable difference in the MTM_TE group. The forms of MT used were effective in reducing the patients’ pain intensity; however, a significant difference between therapies occurred after 4 treatments (*p* = 0.0001). Analyzing the MID between methods, it was observed that self-therapy had an analgesic effect only after 8 treatments, while PIR after 3 and massage after 1 treatment. After day 7, the mean pain score in the MTM_TE group was 0.889 and in the TMPIR_TE group was 3.44 on the NRS scale. In terms of MMO, a significant difference was obtained between monotherapy and each form of TM, i.e. massage (*p* = 0.0001) and PIR (*p* = 0.0001). Analyzing mandibular lateral movements, the authors got a significant difference in the proposed MT forms, of which massage treatments exceeded the effectiveness of PIR.

**Conclusions:**

Soft tissue manual therapy and therapeutic exercise are simple and safe interventions that can potentially benefit patients with myogenic TMDs, with massage showing better analgesic effects than PIR.

**Supplementary Information:**

The online version contains supplementary material available at 10.1186/s13005-023-00385-y.

Temporomandibular joint disorders (TMDs) may affect quality of life in an unprecedented manner. These might include, but are not limited to, being primary sources of chronic pain, impairment of chewing, swallowing threshold, speech, and breathing function, gradually becoming one of the major public health problems [1, 2]. Thus, TMDs are not associated with temporomandibular joints (TMJ) and masticatory muscles alone. What is more, they are often intertwined with other health issues affecting the head and neck region, such as headache, ear symptoms, and cervical spine dysfunction [3, 4].

According to epidemiological data, a large proportion of the world’s population is affected by TMDs, with an estimated 25% of adults presenting with physical and/or visible symptoms [5]. TMDs affect 1.5 to 2.5 times more women than men [6].

In recent years, there has been a significant development in the knowledge of the etiology and treatment of the aforementioned problems. With the continuous search for better diagnostic and therapeutic approaches, attention has been directed at the possibility of using non-invasive therapeutic strategies in patients with TMDs symptoms [7]. In particular, the collaboration between dentist and physiotherapist helps to settle a diagnosis early on and greatly improves the accuracy and effectiveness of therapeutic interventions to follow. Thanks to steady, yet tangible scientific advances, and greater accessibility to specialized therapeutic services, physiotherapy (PT) for TMDs is becoming more and more popular in the medical world. The numerous studies conducted by top physiotherapists/manual therapists such as Mariano Rocabado, Harry von Piekartz and others, have established dental physiotherapy as one of the most dynamic and promising specialties in physical therapy [8, 9].

In daily clinical practice, it can be observed that TMD treatment is usually carried out by a team using several types of interventions, including splint therapy [10], physiotherapeutic techniques such as transcutaneous electrical nerve stimulation (TENS) [11], lasers [12], soft tissue therapies [13], joint mobilization and therapeutic exercise (TE) [14] and psychotherapy [15]. As a conservative treatment, PT is increasingly recognized as an essential part of multimodal approach in TMD management. It has long been used to treat musculoskeletal disorders because it reduces pain and muscle spasms and improves mobility and muscle strength over time and course of sessions [16]. In the daily PT practice, it is considered essential to educate the patient of the causes and consequences of the resulting complaints and disorders. In addition to patient education, implementing home self-therapy into the improvement and self-awareness program is a key. This consists of the systematic performance of therapeutic exercises (TE) individually selected to meet the patient’s condition. Thanks to such management, the patient actively and consciously participates in the healing process and is taught responsibility for their own health.

Numerous studies have evaluated the efficacy of TE in various subacute and chronic musculoskeletal pathologies [17–19], and TE is often recommended for TMDs in combination with manual therapy (MT) [20]. However, the efficacy of these therapies has been studied in the short and medium term, with varying results [21]. Based on systematic reviews, MT, TE, and postural re-education have effectively reduced pain and improved function in TMD patients [22, 23].

The main goals of PT in TMDs are to reduce pain, lower hypertonic muscle hyperactivity and improve function in hypotonic muscles, restore TMJ joint mobility, which eventually leads to mandibular proprioception and biomechanics enhancement. PT treatment is usually reversible and non-invasive [24]. PT methods generally include physical techniques (laser, ultrasound, currents, heat and cold therapy), MT (soft tissue therapy, joint mobilizations, massage) and therapeutic exercises [25]. MT and therapeutic exercise in PT interventions are increasingly being used by clinicians and researched due to positive outcome in most conditions, especially in acute, myofascial and referred pain [26].

According to the contemporary definition given by International Federation of Orthopaedic Manipulative Physical Therapists (IFOMPT), MT should be understood as: “a specialized area of PT dedicated to the management of neuromusculoskeletal diseases, based on clinical reasoning and the application of highly specialized treatment methods, including manual techniques and therapeutic exercises” [27]. MT has been divided into two branches of development. The first ‘soft’ is dedicated to the treatment of soft tissues. This includes post-isometric muscle relaxation (PIR), deep tissue massage, myofascial release, transverse massage, positional release, and fascial therapy, among others. The second branch, called ‘joint’ (‘hard’) – focuses on the hard tissues of the body. It is based on performing manipulation, a technique that involves rapidly exceeding the physiological range of movement in a joint, using high speed but with low amplitude [28]. Manual therapy has been used to restore normal ROM, reduce local ischaemia, stimulate proprioception, break fibrous adhesions, stimulate synovial production and reduce pain [29].

According to a study by Rashid et al., up to 72% of respondents found PT to be an effective form of TMD treatment, with the most effective treatments being therapeutic TMJ exercises (79%), ultrasound (52%), MT (48%), acupuncture/dry needling (41%) and laser therapy (15%) [30].

In the literature, there is a growing number of current scientific studies evaluating the effectiveness of PT. On the contrary, the importance of soft tissue MT and therapeutic exercises requires further randomized controlled trials (RCTs), which are the best evidence for interventions [31, 32]. Therefore, the authors of this study attempted to evaluate the effectiveness of soft tissue TM treatments and therapeutic exercises in TMD patients. To determine whether there was a difference in the effectiveness of the selected treatments, it was decided to compare the myorelaxant, analgesic and functional effects of two commonly used TM treatments. Massage and post-isometric muscle relaxation (PIR).

Most scientific findings emphasize the effectiveness of masticatory muscle massage in the treatment of soft tissue disorders, achieving both muscle relaxation, improved tissue blood supply and joint range of motion and reduced pain [33]. PIR is one of the most well-known mobilization techniques using muscle excitation and inhibition phenomena. It reduces the tension of a muscle or even an entire muscle group, as it inhibits the motoneuron field of a given muscle and thus leads to reflex relaxation [34]. The reason for this is the activation of the Golgi tendon organs during contraction. There are 2 PIR targets – short-term and long-term. The immediate goal is primarily to alleviate pain and other effects of static muscle overload and to reduce muscle and connective tissue irritation. On the other hand, the long-term goal is to restore the expected length and flexibility of contracted muscles, regain normal joint range of motion decreasing potential overload of synovial tissue. As a result, post-isometric muscle relaxation is effective in, among other things, treating increased tension and reducing TrPs. It is now widely used in everyday clinical practice for both musculoskeletal therapy and TMDs [34].

Therefore, we hypothesized that MT factors and self-therapy alter the bioelectrical function of the masseter muscle, possibly contributing to improved TMJ mobility. A reduction in muscle tension results in a decrease in pain intensity.

This study investigates the effectiveness of applied manual factors and therapeutic exercises in eliminating pain and increased muscle tension in female patients with TMDs.

## Material and methods

The study was conducted on female patients from the University Dental Clinic of the Pomeranian Medical University in Szczecin, Poland. Patients (Polish women living in the West Pomeranian Voivodeship) between 20 and 45 years of age (median 28.1) diagnosed with myofascial pain with limited mouth opening (Ib) based on DC/TMD criteria were included in the study.

Exclusion criteria included:

1. Inflammation in the oral cavity that emerged as myospasm or preventive muscle contraction,
2. Earlier splint therapy—could affect the value of the amplitude in the EMG examination, among other variables throughout a signal acquisition procedure.
3. Pharmacotherapy (e.g., oral contraception, hormone replacement therapy, and antidepressants) – some hormones and their replacements are known to affect muscle tone and pain intensity;
4. Systemic diseases (e.g., rheumatic and metabolic diseases)—they can affect muscle tone and pain intensity and range of motion TMJ
5. Mental illness- they can affect muscle tone and pain intensity, both treated and untreated
6. Lack of stability in the masticatory organ motor system—this affects muscle tone and pain intensity and range of motion TMJ
7. Masticatory organ injury—can affect muscle tone and pain intensity and range of motion TMJ, usually due to myospasm/local myalgia/preventive co-contraction
8. Pregnancy – as trimester-dependent estrogen/progesterone and relaxine interplay may affect muscle tone and pain intensity,
9. Patients undergoing orthodontic treatment—can affect muscle tone and pain intensity and range of motion TMJ,
10. Other types of inflammation in the oral cavity (e.g., pulp inflammation or impacted molars) – which usually yield in protective co-contraction,
11. Fibromyalgia—can affect muscle tone, pain intensity and/ as well as range of motion in TMJ and cervical spine,
12. Other specific contraindications for use of physical treatments in the MT, e.g. cancer therapy, some older models of artificial pacemakers, etc.

**Fig. 1:**
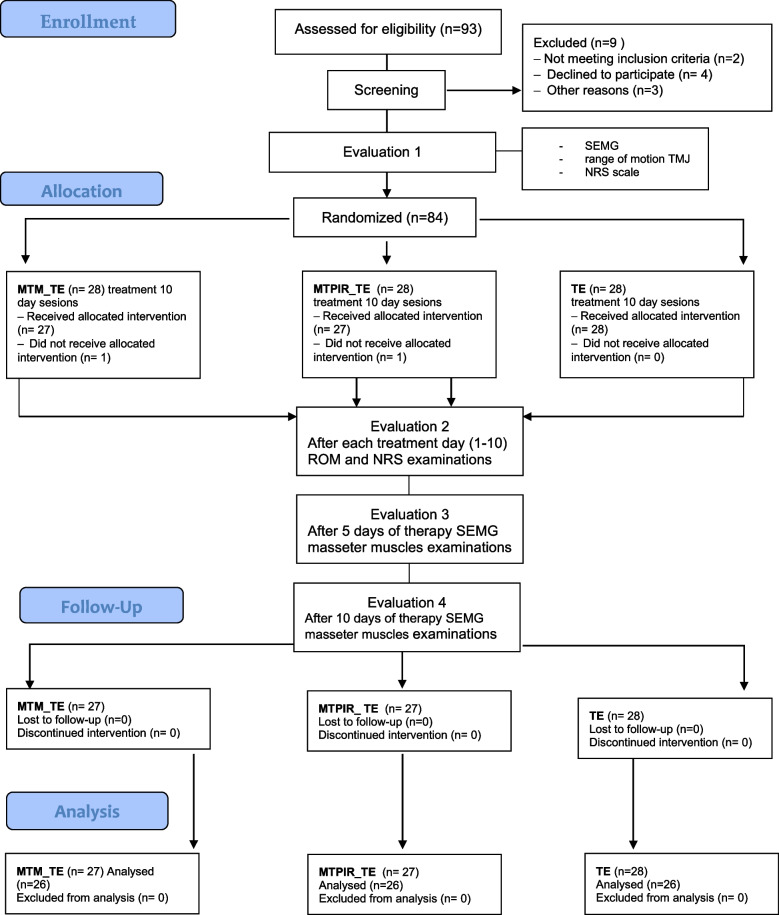
CONSORT flowchart of the participants’ progress through the trial phases [35]

All women underwent an intra-oral and extra-oral dental examinations performed by orofacial pain trained dentist. The aim was to exclude odontogenic, periodontal and articular causes of TMD pain. Women meeting the above criteria constituted the study group (G1, *n* = 82). The patients qualified for the study underwent instrumental diagnostics (sEMG of the masseter muscles at rest and exercise, linear measurement of the range of mandibular mobility) and the level of pain intensity was assessed on the NRS numerical scale (Fig. 1).

The control group (G2, *n* = 104) consisted of healthy women Patients (Polish women living in the West Pomeranian Voivodeship), aged 20 to 45 (median 29), without claimed TMDs and pain disorders (based on the extra-oral and intra-oral dental examination, DC/TMD criteria, NRS scale), in whom SEMG tests were performed, and TMJ mobility measurements were performed.

The research project was approved by the Bioethics Committee of the Pomeranian Medical University in Szczecin (no. KB – 0012/102/13). Information on the clinical trial registration is available at www.ClinicalTrials.gov (NCT05021874). All participants gave their written consent. The study complies with the CONSORT guidelines for reporting randomized controlled trials [27].

### sEMG test of masseter muscle

sEMG recordings from the masseter muscles were performed with a two-channel NeuroTrac MyoPlus 2 device with NeuroTrac software (Verity Medical Ltd., Tagoat, Ireland). Clinical Mode EMG was used during the study. To obtain precise sEMG measurements, a band-stop filter was used, which guarantees that the frequencies of 50 Hz and 60 Hz (mains) will not interfere with the recording of muscle activity (measured in microvolts). Specialized filtering allows sEMG to be measured with a precision of 0.1 µV.

In order to avoid magnetic interference, when collecting sEMG measurements, the device was not placed in the vicinity of cell phones (< 4 m) or other sources that might interfere with the results. Two unipolar electrodes were used for the test, which were attached at a distance of 10 mm from each other. The main principle used in all subjects was to position the electrodes over the center of muscle belly, parallel to the path of its fibers (the lower electrode was placed approx. 5 mm above the mandibular angle and the upper electrode 10 mm above it); precise placement of the electrodes was preceded by careful palpation of each muscle. The masseter muscles bioelectrical signals were acquired in an upright sitting position, with the head in a natural, postural position, hands resting on the knees and feet resting on the ground. Before the electrodes were applied, the skin was cleaned with rubbing alcohol disinfectant, following electrode manufacturers’ manual and guidelines of Seniam project (www.seniam.org). The neutral electrode was located on the cervical section—C7 styloid process, which is usually devoid of vastly active muscle fibers.

- Examination of the electrical activity of the masseter muscle at rest (Rest Test): the test was performed on relaxed and relaxed patients. The dental arches remained slightly open during the examination. In order to eliminate the registration of signals related to the electrical activity of the eye circular muscle, these persons had their eyelids closed during the measurements. The patients were instructed not to swallow saliva during the examination and to place their tongue in a resting position.
- Study of the bioelectrical activity of the masseter muscle during maximal muscle contraction (MVC): sEMG signal was recorded in a sitting position, while clenching the teeth, using the greatest possible force, within 5 s. The computer program with which the device cooperated registers the minimum and maximum values and calculates the average values of electric potentials.

The sEMG values obtained were normalized as the ratio of RLX to MVC.

Activity normalized to MVC [%] = RLX [µV]/ MVC [µV] × 100% [28].

Mean sEMG values were then calculated between the right and left masseter muscles.

The G1 patients were then randomly divided into three treatment subgroups, in which physiotherapy management was implemented over a period of 10 days (excluding Saturdays and Sundays). Two subgroups received manual therapy and therapeutic exercises/massage—MTM_TE; PIR—MTPIR_TE), while the third subgroup was prescribed monotherapy—TE.

In women with G1, the pain was assessed each time after therapy using the NRS scale, and the mandibular range of motion was evaluated. After the 5th and 10th therapeutic day, a follow-up sEMG examination was performed at rest and on exertion.

## Therapeutic interventions

In the therapeutic management, behavioral therapy was implemented first in all subjects, which consists of explaining the causes of the dysfunctions and ways of self-control of occlusal and non-occlusal parafunctions, especially in the form of clenching and grinding of teeth or excessive gum chewing. Great importance is placed on the patient’s cooperation in the treatment process. Patients were informed about the essence of self-monitoring of parafunctions, the pathophysiology of the dysfunction and the impact of the patient’s own therapeutic cooperation on the effectiveness of treatment. The PIR treatments were performed in the group MTM_TE and the massage treatments were performed in the MTPIR_TE group. A standard exercise regimen was tailored to all participants. The description of the intervention is included in Supplement 1.

### Statistical analysis

The data were presented as a median with a quartile range due to significant deviations from the normal distribution assessed using the graphical method with the analysis of the histogram and the Q-Q plot. Due to significant deviations from the normal distribution, the dependence of the studied variables on time and between groups was performed based on the nparLD test, which is a non-parametric equivalent of the analysis of variance for repeated measurements. The Kruskall-Wallis test with the post-Hoc multiple comparison test was used to compare the baseline values of the parameters studied with the control group. Comparisons between the control group and the total study group were made using the U-Mann Whitney test. The values of *p* < 0.05 were considered significant. The analysis was performed in the R language in the RStudio software using the NparLD, ggplot2, ggpubr libraries. Minimal important difference was defined as 0.5 × SD for each Variable [36, 37].

## Results

The results from the pre-test in the test and control group are presented first. The results from the sEMG study represent the mean value between the right and left masseter muscle.

There was no difference in the age structure between the groups. In the diagnostic procedure, between G1 and G2, statistical differences were found in the sEMG value of the masseter muscle (RLX test, MVC, MVC%), all ranges of TMJ mobility and pain intensity (Table 1). The minimal important difference (MID) values are presented in Table 2.

**Table 1:** Statistical analysis of the age structure, sEMG values, TMJ range of motion and pain intensity in the G1 (study group) and G2 (control group) at baseline

Variable	G1 *n* = 82			G2 *n* = 104			p
	Me	Q1	Q3	Me	Q1	Q3	
Age	28.1	23	38	29	25	39	0.24
MMO [mm]	36	35	37	45	44	46	0.00
RLM [mm]	6	6	7	10	9	10	0.00
LLM [mm]	6	5	7	10	9	11	0.00
NRS scale	6	6	7	0	0	0	0.00
sEMG (RLX Test) [µV]	35.3	28.45	42.65	1.72	1.3	3.44	0.00
sEMG (MVC) [µV]	283.5	231	343.5	66.9	59.55	76	0.00
sEMG (%MVC)	15.25	11.02	18.15	2.55	1.83	3.3	0.00

*Legend: MMO* Maximal Mouth Opening, *RLM* Right lateral movement, *LLM* Left lateral movement, *NRS* Numeric Rating Scale, *RLX test* Rest test, *MVC* Maximum Voluntary Contraction

**Table 2:** Minimal important difference in the G1 (*n* = 82)

Variable	Mean	SD	MID
MMO [mm]	36.2	1.48	0.7
RLM [mm]	6.18	0.877	0.4
LLM [mm]	6.06	0.934	0.5
NRS scale	6.2	0.987	0.5
sEMG (RLX Test) [µV]	36	7.3	3.7
sEMG (MVC) [µV]	290	74.8	37.4
sEMG (%MVC)	15.3	5.33	2.7

*Legend: MID* Minimal important difference, *MMO* Maximal Mouth Opening, *RLM* Right lateral movement, *LLM* Left lateral movement, *NRS* Numeric Rating Scale, *RLX test* Rest test, *MVC* Maximum Voluntary Contraction

**Fig. 2:**
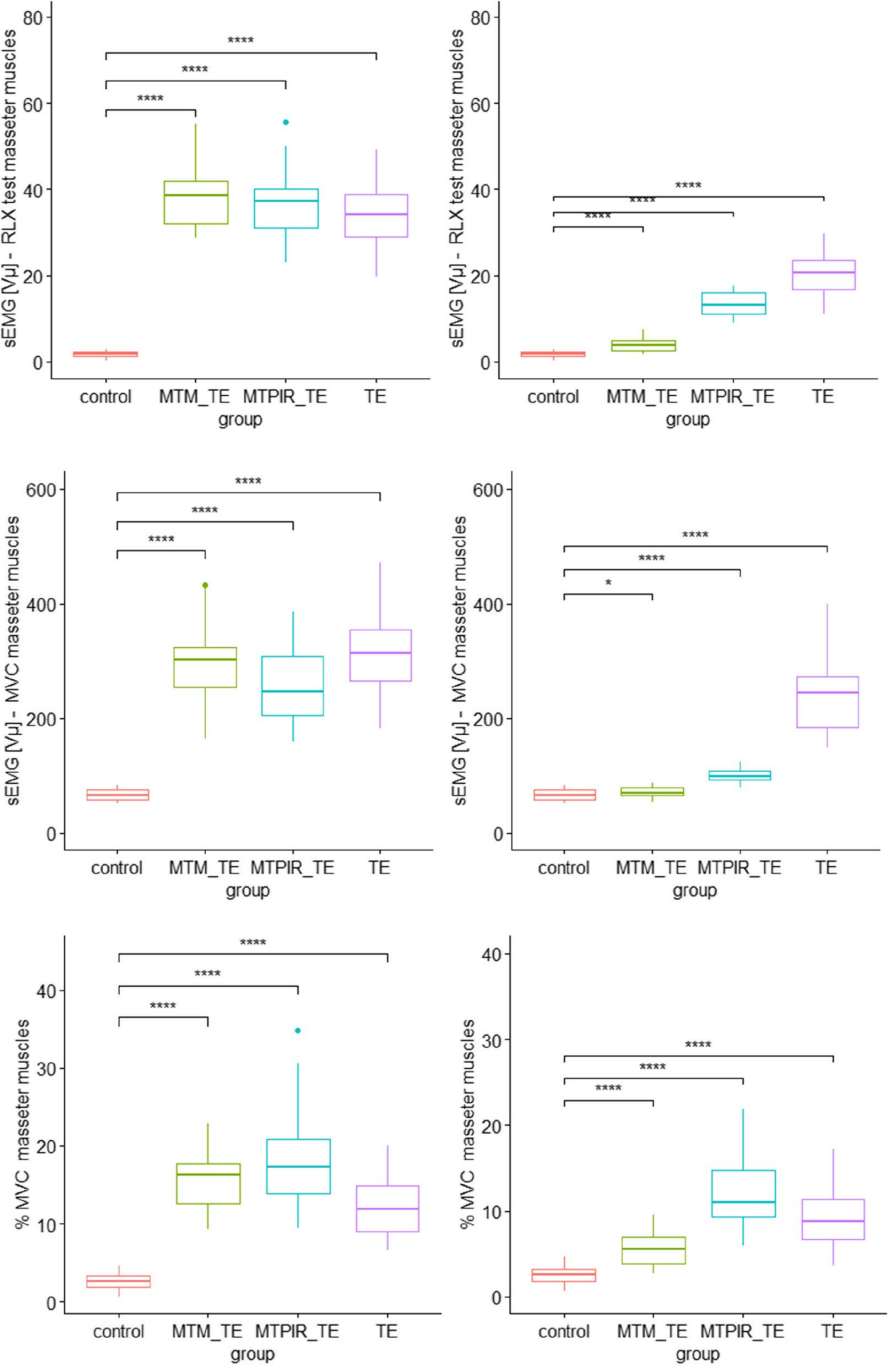
Statistical analysis of the sEMG distribution, in the treatment groups before (left side) and after (right side) therapy and the control group. Legend: MVC- Maximal Voluntary Contraction; RLX test – Rest test; MTM_TE – manual therapy (massage) and therapeutic exercises; MTPIR_TE – manual therapy (PIR) and therapeutic exercises; TE – therapeutic exercises; control – control group. symnum.args a list of arguments to pass to the function symnum for symbolic number coding of *p*-values. For example, symnum.args <—list(cutpoints = c(0, 0.0001, 0.001, 0.01, 0.05, 1), symbols = c(“****”, “***”, “**”, “*”, “ns”)). In other words, we use the following convention for symbols indicating statistical significance: ns: *p* > 0.05*: *p* <  = 0.05; **: *p* <  = 0.01; ***: *p* <  = 0.001; ****: *p* <  = 0.0001

**Fig. 3:**
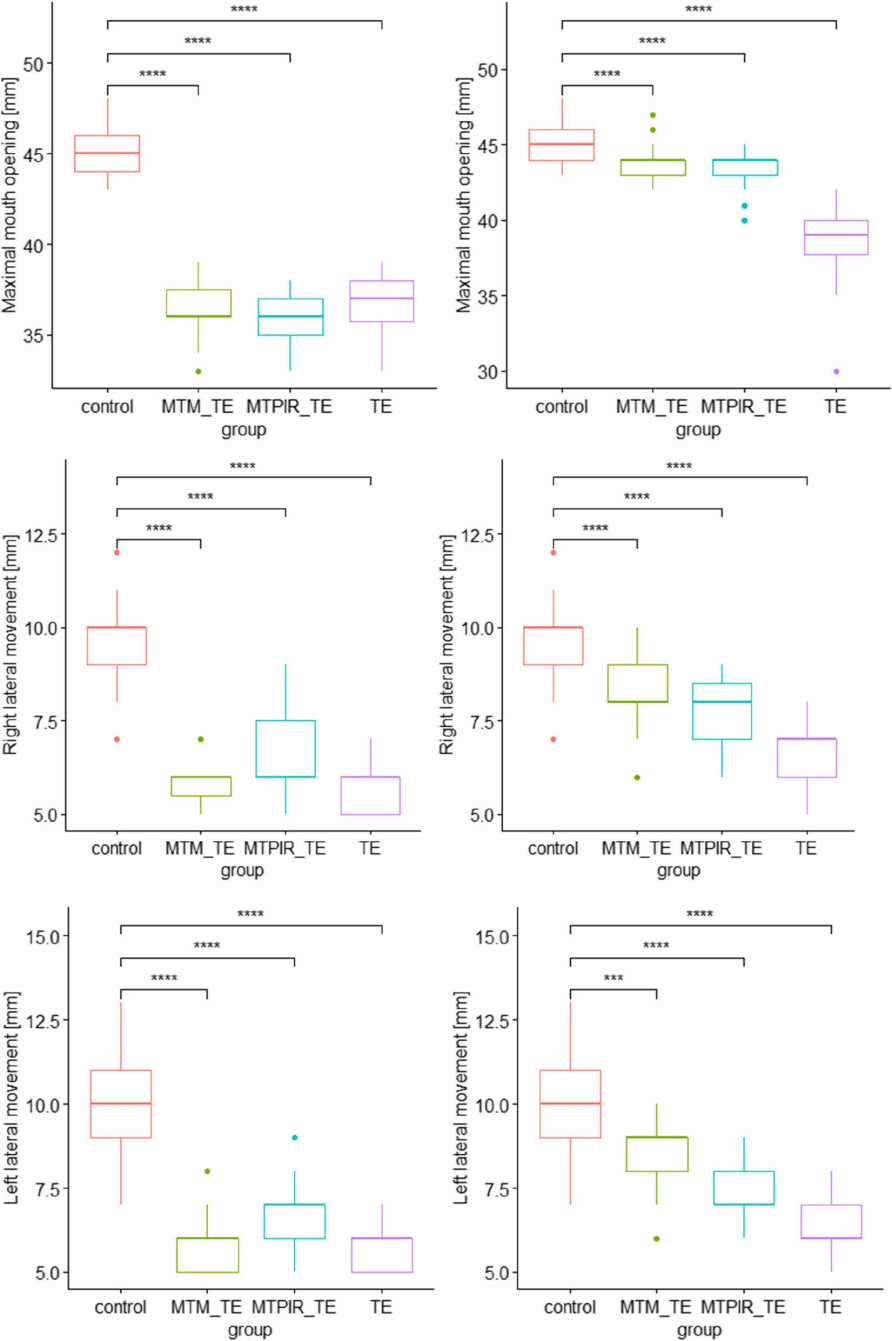
Statistical analysis of the mandibular mobility range in the treatment groups before (left side) and after (right side) therapy and the control group. Legend: MTM_TE – manual therapy (massage) and therapeutic exercises; MTPIR_TE – manual therapy (PIR) and therapeutic exercises; TE – therapeutic exercises; control – control group. symnum.args a list of arguments to pass to the function symnum for symbolic number coding of *p*-values. For example, symnum.args <—list(cutpoints = c(0, 0.0001, 0.001, 0.01, 0.05, 1), symbols = c(“****”, “***”, “**”, “*”, “ns”)). In other words, we use the following convention for symbols indicating statistical significance: ns: *p* > 0.05*: *p* <  = 0.05; **: *p* <  = 0.01; ***: *p* <  = 0.001; ****: *p* <  = 0.0001

**Fig. 4:**
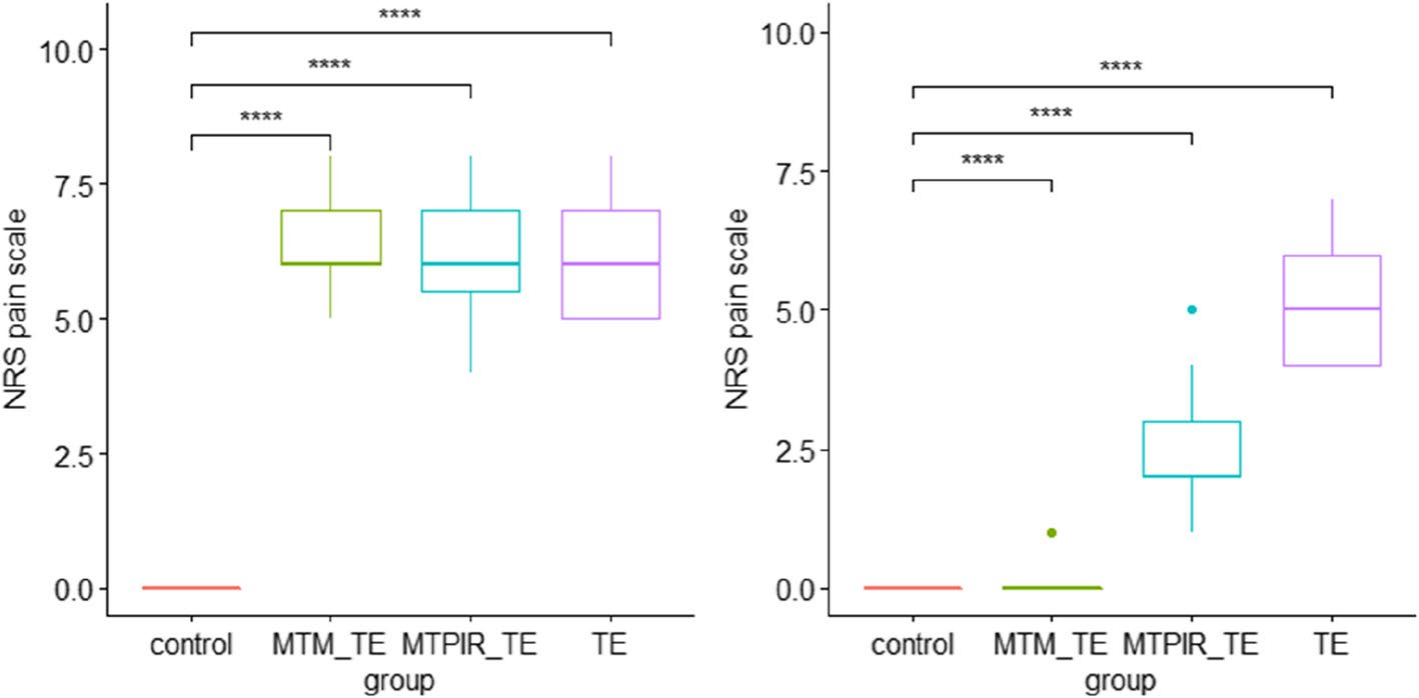
Statistical analysis of the pain intensity in the treatment groups before (left side) and after (right side) therapy and the control group. Legend: NRS- Numeric Pain Rating Scale; MTM_TE – manual therapy (massage) and therapeutic exercises; MTPIR_TE – manual therapy (PIR) and therapeutic exercises; TE – therapeutic exercises; control – control group. symnum.args a list of arguments to pass to the function symnum for symbolic number coding of *p*-values. For example, symnum.args <—list(cutpoints = c(0, 0.0001, 0.001, 0.01, 0.05, 1), symbols = c(“****”, “***”, “**”, “*”, “ns”)). In other words, we use the following convention for symbols indicating statistical significance: ns: *p* > 0.05*: *p* <  = 0.05; **: *p* <  = 0.01; ***: *p* <  = 0.001; ****: *p* <  = 0.0001

The following (Figs. 2, 3 and 4) present a comparative analysis of variables (sEMG, MMO, LLM, RLM, NRS) in therapeutic subgroups after (left side) and before (right side) therapy and in the control group.

Significantly higher scores were observed in all sEMG measurements in each treatment subgroup compared to the control group (*p* <  = 0.0001). PT interventions in each subgroup (MTM_TE; MTPIR_TE; TE) were followed by statistically significant changes in the parameter studied, with TM treatments showing a better effect than monotherapy (Fig. 2).

Mandibular movement ranges in the control group were significantly higher than in the study groups (*p* <  = 0.0001). Physiotherapeutic interventions were followed by statistically significant changes in each of the subgroups (MTM_TE; MTPIR_TE; TE) for the parameter studied, with TM treatments showing a better effect than monotherapy (Fig. 3).

After physiotherapeutic interventions, in each of the subgroups (MTM_TE; MTPIR_TE; TE) there were statistically significant changes in the examined parameter, with TM treatments showing a better effect compared to monotherapy. Only the treatment with the use of massage led to complete pain relief (Fig. 4).

**Fig. 5:**
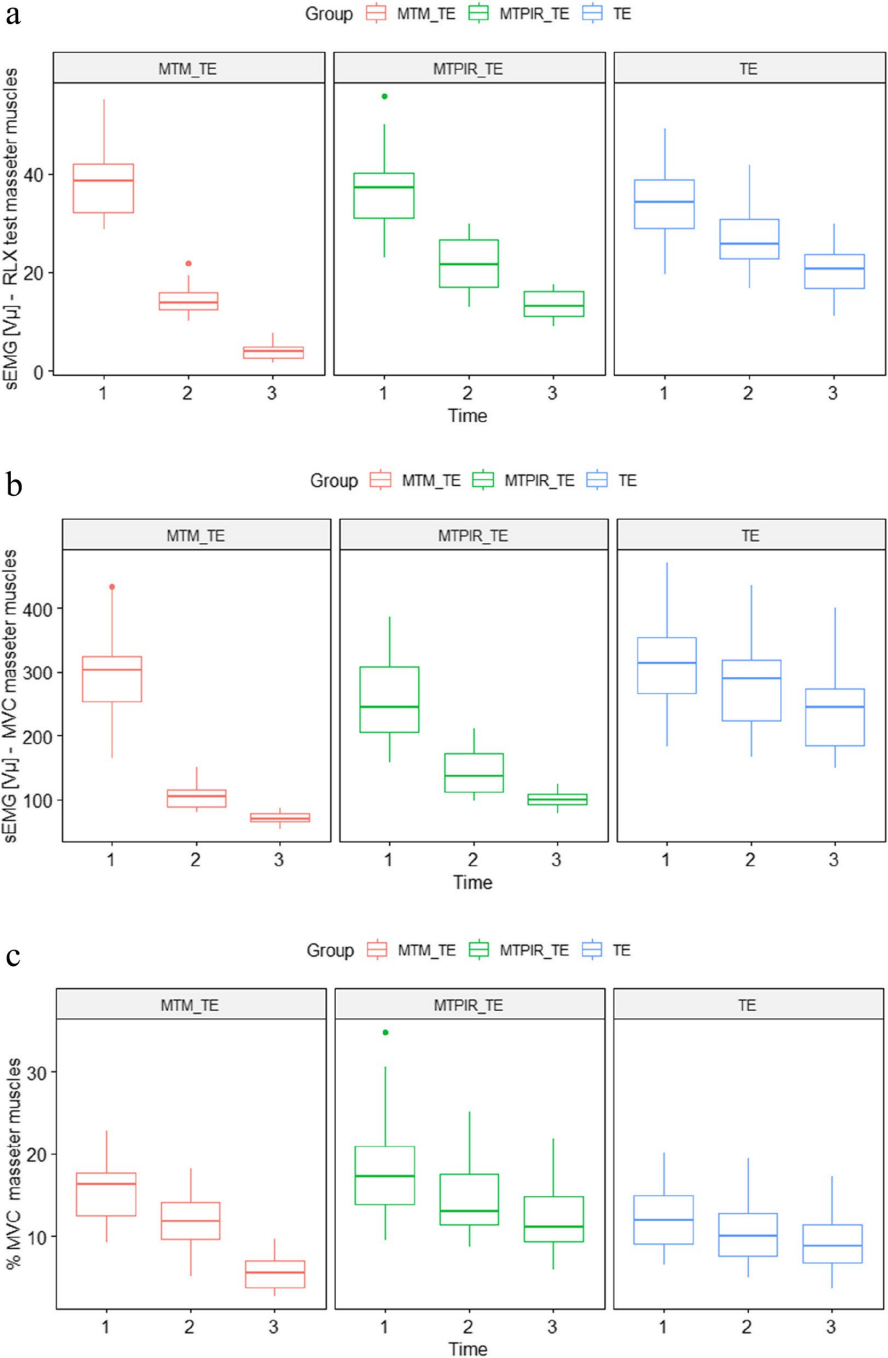
**a** sEMG Rest test analysis in the G1 group before, during and after the end of the therapies. p_group, p_time p_time*group *p* < 0.001. Legend: Rest test; MTM_TE – manual therapy (massage) and therapeutic exercises; MTPIR_TE – manual therapy (PIR) and therapeutic exercises; TE – therapeutic exercises. **b** sEMG MVC in the G1 group before, during and after the end of the therapies. p_group, p_time p_time*group *p* < 0.001. Legend: MVC- Maximal Voluntary Contractions; MTM_TE – manual therapy (massage) and therapeutic exercises; MTPIR_TE – manual therapy (PIR) and therapeutic exercises; TE – therapeutic exercises. **c** sEMG analysis (MVC%) in the G1 group before, during and after the end of the therapies. p_group, p_time p_time*group *p* < 0.001. Legend: %MVC-% Maximal Voluntary Contractions; RLX test – Rest test; MTM_TE – manual therapy (massage) and therapeutic exercises; MTPIR_TE – manual therapy (PIR) and therapeutic exercises; TE – therapeutic exercises

Figure 5 a-c below shows the effect of the applied physiotherapeutic treatments in all four therapeutic subgroups on sEMG during the initial study (1), after 5 days of therapy (2), and after the 10th treatment day (3).

The significant interaction effect of time and method indicates the highest effectiveness of MTM_TE among the methods tested (5a-c).

**Fig. 6:**
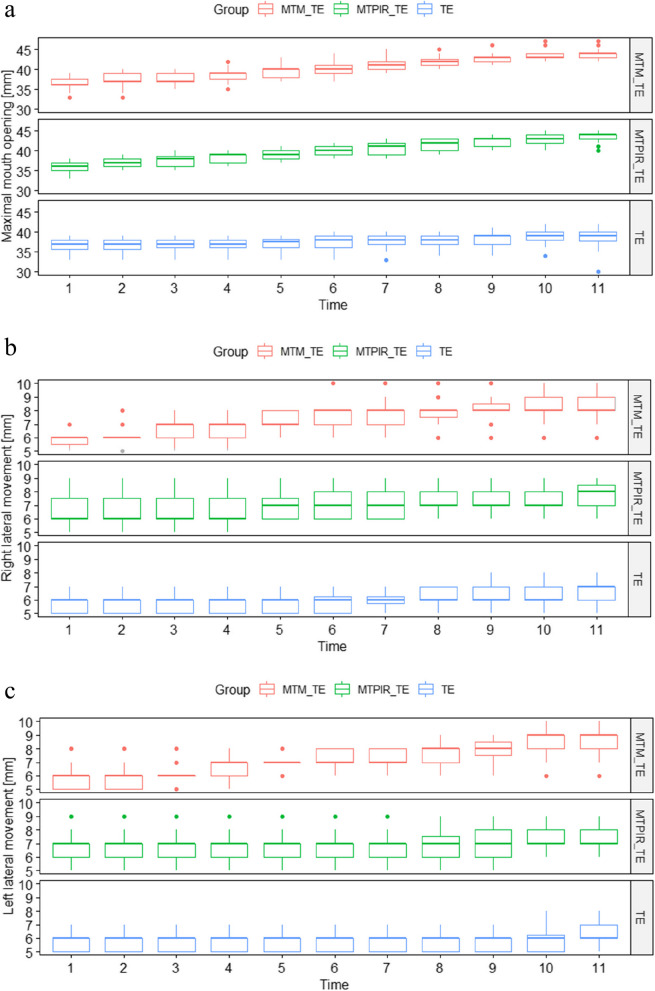
**a** Analysis of the maximal mouth opening in the G1 group before, during and after the therapies. Legend: MTM_TE – manual therapy (massage) and therapeutic exercises; MTPIR_TE – manual therapy (PIR) and therapeutic exercises; TE – therapeutic exercises. **b** Analysis of the range of right lateral movements in the G1 group before, during and after the therapies. Legend: MTM_TE – manual therapy (massage) and therapeutic exercises; MTPIR_TE – manual therapy (PIR) and therapeutic exercises; TE – therapeutic exercises. **c** Analysis of the range of left lateral movements in the G1 group before, during and after the therapies. Legend: MTM_TE – manual therapy (massage) and therapeutic exercises; MTPIR_TE – manual therapy (PIR) and therapeutic exercises; TE – therapeutic exercises

Figure 6 a-c shows the 10-day analysis (1—initial examination, 11—tenth day of therapy), the range of mandibular mobility in three therapeutic subgroups.

The significant interaction effect of time and method indicates the highest effectiveness of manual therapy among the methods studied (6a-c).

**Fig. 7:**
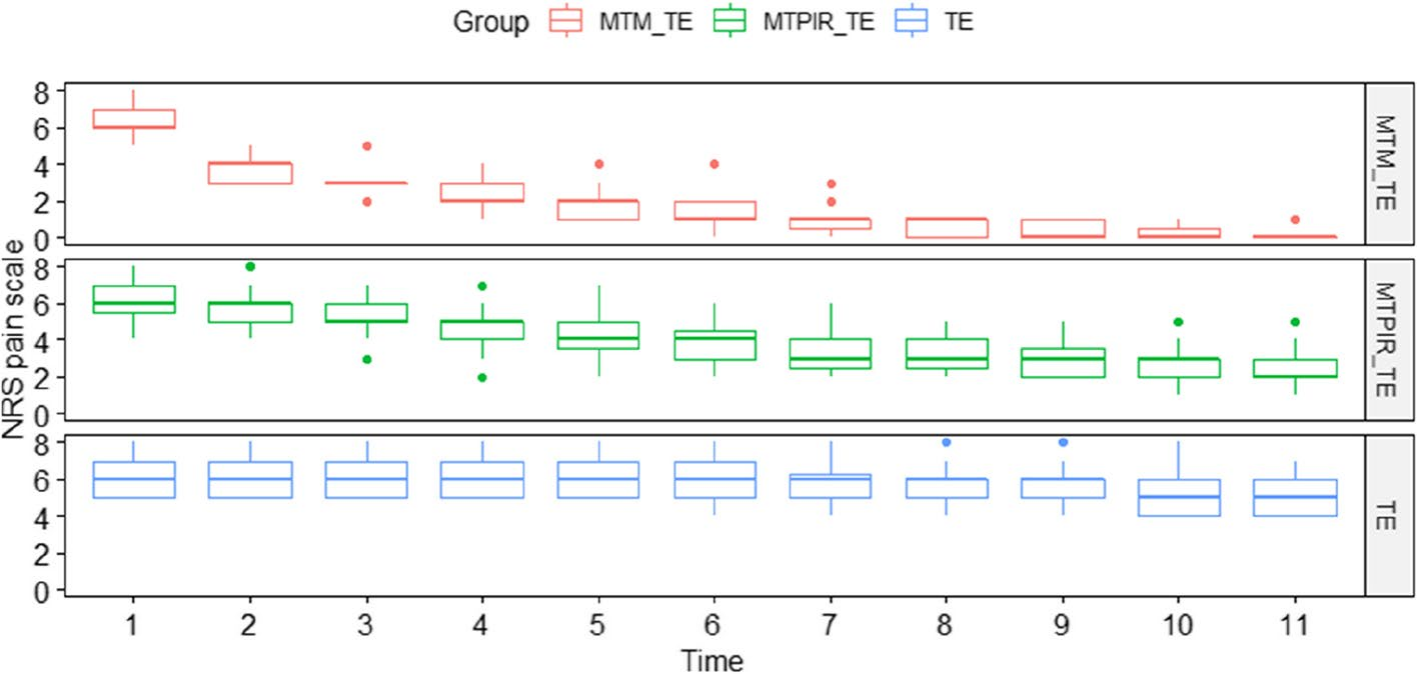
Analysis of the pain intensity in the G1 group before, during and after the therapies. Legend: NRS – Numeric Rating Scale; MTM_TE – manual therapy (massage) and therapeutic exercises; MTPIR_TE – manual therapy (PIR) and therapeutic exercises; TE – therapeutic exercises

Figure 7 shows the 10-day analysis (1—initial examination, 11—tenth day of therapy), the intensity of pain on the NRS scale.

The significant interaction effect of time and method indicated that manual therapy was the most effective among the methods studied. Of these, MTM_TE proved to be the most effective method for pain alleviation (Fig. 7).

As shown in Table 3, each of the treatments led to different changes in parameters during the study.

**Table 3:** Comparison of differences between parameters in the course of the study by group

Variable	MTM_TE			MTPIR_TE			TE			*P*
	Me	$\overline{x}$	SD	Me	$\overline{x}$	SD	Me	$\overline{x}$	SD	
d_SEMG mean RLX [µV]	-28.7	-26.6	8.41	-18.1	-18.6	8.4	-11.4	-11.8	4.28	< 0.001
d_SEMG mean MVC [µV]	-230	-222	71.8	-152	-159	59	-64.5	-71.7	32.5	< 0.001
d_SEMG mean %MVC	-10.4	-9.79	3.32	-6.13	-6.55	3.8	-2.81	-2.84	0.933	< 0.001
d_MMO [mm]	7	7.37	1.44	8	7.7	1.73	2	2.04	2.03	< 0.001
d_RLM [mm]	3	2.59	0.694	1	0.963	0.706	1	0.893	0.315	< 0.001
d_LLM [mm]	3	2.74	1.02	1	0.926	0.474	1	0.643	0.488	< 0.001
d_NRS	-6	-6.07	0.997	-4	-3.82	1.39	-1	-0.964	0.429	< 0.001

*Legend: MTM_TE* Manual therapy (massage) and therapeutic exercises, *MTPIR_TE* Manual therapy (PIR) and therapeutic exercises, *TE* Therapeutic exercises

## Discussion

This study determined the analgesic and myorelaxant efficacy of MT and self-therapy treatments in female patients with TMDs. The bioelectrical function of the masseter muscles and the evaluation of mandibular movements in patients with and without TMDs were also scrutinized.

When analyzing the bioelectrical function of the masseter muscle at rest and exercise, the study’s authors observed a significant difference between the control group and those with TMDs (*p* = 0.00). As can be seen from the diagnostic part of the study, subjects with TMDs have higher mean sEMG values of the masseter muscle at rest and during exercise. The increased bioelectrical activity of the masseter muscle is considered to influence the restriction of mandibular MMO ranges and lateral mandibular movements in the study group. As can be seen from the analysis, the mean pain intensity before PT was 6 (moderate pain) on the NRS scale. As shown by the results of studies conducted by other authors, increased muscle tension affects the range of mobility of the TMJ and contributes to pain [38].

In the treatment of patients with TMDs, an interdisciplinary treatment team consisting of, among others, an Orofacial Pain-trained dentist/pain physician specializing in TMJ treatment (prosthetics specialist, orthodontist), physiotherapist, psychologist and psychiatrist is key. When prescribing treatment, the first therapy recommended for a patient with pain and impaired joint function is MT combined with self-therapy and supplemented with behavioral therapy. Calixtre et al., in a systematic review of RCTs about studies on the effectiveness of MT in patients with TMDs, indicated that soft tissue therapy treatments applied to the masticatory muscles are even more effective than controls but as effective as botulinum toxin injections [39]. They concluded that there is very different evidence that MT relieves pain, MMO and pressure pain in people with signs and symptoms of TMDs, depending on the technique. Therefore, further RCTs evaluating the effectiveness of specific MT treatments in patients with TMDs should be conducted. Therefore, to fill this gap in the literature, the authors of this study attempted to evaluate the effectiveness of the two most commonly used soft tissue MT treatments, i.e. massage and PIR in TMD patients.

As can be seen from the study, each of the prescribed forms of physiotherapy altered the bioelectrical function of the masseter muscles, the range of mandibular mobility and the intensity of pain. Analyzing the individual parameters studied in relation to the proposed forms of therapy, it can be concluded that there were significant differences between the study groups.

Both massage, PIR and self-therapy led to a decrease in bioelectrical signals of the masseter muscle at rest as well as in exercise. When analyzing this parameter, the authors observed that no significant difference was obtained at the first time point between the TMM_TE, PIT_TE, and TE groups (*p* > 0.05). Only at the 2nd time point (after 5 treatments) was a significant difference obtained between the groups (*p* = 0.0001). The result obtained may indicate the need for at least 5 TM treatments to improve the bioelectrical function of the muscle. Analyzing the above parameter at the time endpoint (i.e. after 10 treatments), it can be concluded that, of the proposed physiotherapeutic treatments, massage combined with TE (MTM_TE) proved to be the most effective form of therapy in patients with TMDs and outperformed in its effectiveness TEPIR_TE (*p* = 0.0001) and TE (*p* = 0.0001). The result obtained may indicate that muscle massage treatments have a more substantial effect on masticatory muscle activity. Each proposed therapy showed a minimal clinically significant difference in the sEMG parameter at this endpoint. The largest MID is seen in the TMM_TE group in both resting and exercise muscle activity. The smallest MID occurred in the TE group, and in exercise muscle function, this only occurred at the second time point (after 5 treatments). Single scientific reports similarly compare the effectiveness of TE, TM, and PIR in combination with self-therapy. A study by Urbanski et al. conducted on patients with TMDs found no significant difference, in terms of myorelaxant effect, between PIR and muscle-fascial therapy. In both groups, a decrease in masseter and temporalis muscle SEMG function was observed, as well as a reduction in pain intensity. The differences between the study results may be due to the use of a different methodology for conducting the soft tissue therapy procedure in the above study [40].

Ariji et al. conducted an ultrasound study of masseter muscle features as an indicator to assess the effectiveness of massage. After the massage, the thickness of the muscle on the symptomatic side decreased significantly, the anechoic areas decreased by 85%, and the pain intensity decreased with it. According to their study, the thickness of the masseter muscle and the presence of anechoic areas may be related to the therapeutic efficacy of muscle pain [41].

We also noticed that the MT forms used effectively reduced the patients’ pain intensity. Notably, a significant difference between the therapies occurred at the second time point (after the 4th treatment). This analgesic effect may occur due to MT pain modulation via activating low-threshold Aβ fibers pathway. This inhibits nociceptive stimuli from Aδ and C supply fibers [42, 43]. MT can also elicit affective responses activating opioid, oxytocin and dopaminergic pathways [44, 45]. As TMDs are disorders involving the masticatory muscles, TMJ and related structures [44], it has been hypothesized that MT applied to craniomandibular structures positively affects pain perception [45]. However, it is essential to note that the quality of evidence supporting this hypothesis is still low and requires further research, although the available evidence supports the use of MT for pain relief in TMD patients.

When assessing the analgesic effect of MT, the authors observed that, again, the most effective form of pain management was massage combined with autotherapy, which was superior to the other two treatment groups (*p* = 0.0001). Analyzing the minimal clinically significant difference between the methods, it was observed that auto-therapy had an analgesic effect only after 8 treatments (a decrease of 0.5 points), while in the MTPIT_TE groups after 3 (a reduction of 0.52 points) and MTM_TE after 1 treatment (a mean decrease of 2.63 points). It is noteworthy that, from a clinical point of view, after the 7th day of massage, the mean pain score on the NRS scale was 0.889, and that of the patients subjected to PIR was 3.44. Thus, it can be concluded that, of the proposed forms of physiotherapy, massage combined with TE is the most effective therapy for treating masseter muscle pain. The therapeutic importance of massage has been extensively described in a peer-reviewed publications recently. It is related to many factors, such as local blood and lymph flow, muscle activity and the nervous system [46]. Massage is improving blood flow through small vessels because muscle tension is reduced, leading to better and faster regeneration, oxidative capacity of muscle tissue and improving the range of movement [47]. In addition, massage has analgesic properties (activation of the control gate mechanism) and has sound psychological effects, reducing stress and anxiety and improving the patient’s mood through endogenous cannabinoid/opioid system pathway, however this statement require further studies in larger cohorts [48, 49].

According to a study by Rodrigez Blanco et al., the PIR technique of the masseter muscle proved to be a more effective method than the tension/counter-tension technique in patients with pain and limited mandibular visitation [50]. In contrast, a study by Ibáñez-García et al. found no significant differences (*p* > 0.8) between the PIR method and tension/counter-tension in the treatment of TMJ pain and mobility [51]. Rajadurai et al. demonstrated the effectiveness of muscle energization techniques (METs) involving PIR and reciprocal inhibition in treating pain and improving MMO in patients with TMDs [52].

As shown in a study by Gomes et al. comparing the therapeutic effect of massage and splinting in an RCT in patients with TMDs, massage therapy on the masticatory muscles and the use of an occlusal splint leads to an increase in mandibular ROM similar to that in an asymptomatic comparison group in relation to maximum active mouth opening and right and left movement in TMD patients [53]. On the contrary, in a study by Shousha et al. comparing the therapeutic effect of MT and chiropractic therapy, statistical analysis showed a significant reduction in pain intensity and TMJ opening index between groups in favor of the conservative PT group. Thus, according to their study, over a treatment period of 6 consecutive weeks, conservative PT may be a better initial treatment than occlusal appliances in relieving pain and improving range of motion in cases of myogenous TMDs [54].

Another parameter assessed in this study was TMJ range of motion, which was significantly different in TMDs patients compared to healthy controls (*p* = 0.00).

Statistical analysis showed no significant difference in MMO between the groups at first and second-time endpoints (*p* > 0.5). At other time points (from the 6th treatment onwards), a significant difference was obtained between monotherapy and each form of MT, with both massage (*p* = 0.0001) and PIR (*p* = 0.0001) significantly improving the MMO range. When analyzing the minimum clinically significant difference between methods, it was observed that TE was the least effective (1 point increase on day 7 of therapy). Between the TM methods, the clinical effect was comparable both after day 1 of treatment (TMM_TE increase of 1 point; TMPIR_TE increase of 0.9 points) and at the endpoint (day 10 of therapy—TMM_TE increase of 7.4 points; TMPIR_TE increase of 7.7 points. The results obtained may indicate comparable efficacy of massage and PIR in improving mandibular abduction mobility. However, with regard to mandibular lateral movements, the authors obtained a significant difference in the proposed forms of MT, of which massage treatments were superior in their effectiveness to PIR. Thus, the above results may indicate a greater therapeutic benefit of massaging the masticatory muscles to improve TMJ function. However, these observations require the continuation of the research initiated.

A study by Toucker et al. on the evaluation of MT and therapeutic exercises showed that, when combined, both forms of therapy yield clinically significant effects on both pain and pain-free maximal mouth opening in TMD patients [55]. Also, in the authors’ study, it was observed that synergism of therapeutic agents resulted in better relaxation (*p* = 0.0001) and analgesic effects (*p* = 0.0001) and contributed to a significant increase in TMJ range of motion (*p* = 0.0001) compared to monotherapy.

Herrera-Valencia et al., in their meta-analysis consisting of six papers on the effectiveness of MT in the treatment of TMDs, found that significant improvements in pain and MMO were observed after manual treatment compared to baseline values. According to their analysis, MT appears to be an effective treatment for TMJ disorders in the mid-term, although the effect seems to diminish over time. However, when combined with therapeutic exercise, these effects may persist for a long time [56]. Also, De Laat A et al. demonstrated the effectiveness of a conservative approach, including counselling and physiotherapy (heat, massage, ultrasound and muscle stretching techniques, in improving pain parameters and jaw function in myofascial pain patients [57].

According to Byra et al., the PT procedure used, including MT and masticatory muscle exercises, improves TMJ range of motion and reduces pain in patients with TMJ hypomobility. Significant improvements in TMJ mobility were achieved, with an average increase of 6.6 mm (*p* = 0.0005) and a reduction in pain, with a decrease of an average of three points on the NRS scale (*p* = 0.00002) [58]. The therapeutic efficacy of TM in patients with TMDs and joint hypermobility was also confirmed in similar study by Kulesa-Mrowiecka et al. [59].

The research carried out quite clearly demonstrated the superiority of the synergism of MT methods and therapeutic exercises over monotherapy (self-therapy), at very high significance (*p* = 0.00), which is a major strength of this study. Similarly, is the identification of a minimal clinically relevant difference, whereby it was observed that massage treatments surpassed PIR in their effectiveness, hence producing a better and faster therapeutic effect, especially with regard to pain intensity. The results obtained are auspicious, prompting the authors to continue this type of study in the future.

### Limitations

According to the authors, a limitation of the study carried out was the short duration of therapy the women underwent and the small size of the treatment subgroups. In addition, there was no evaluation of how long the therapeutic effect obtained during the applied treatments persisted after the therapy cycle. Therefore, the authors see a need to continue the study conducted, focusing on the above limitations.

## Conclusions

1. People with TMD should receive at least six manual therapy sessions to improve the bioelectrical function of the masseter muscle, with massage combined with autotherapy appearing to be a more effective therapy than PIR and autotherapy, which should be taken into account when selecting the form of therapy.
2. Massage combined with self-therapy shows a significant analgesic effect in people with the painful form of TMD, leading to a significant or complete resolution of pain after the 7th day of therapy.
3. Massage and post-isometric relaxation of the masseter muscle have comparable efficacy in improving mandibular mobility in patients with TMD.
4. Manual therapy should be part of the therapeutic regimen for patients with TMD.

### Supplementary Information

**Additional file 1: Supplement 1.**

## Data Availability

Data and materials available at request.
