# Supplementary material for: Evaluation of the efficacy of manual soft tissue therapy and therapeutic exercises in patients with pain and limited mobility TMJ: a randomized control trial (RCT)
Source: Head Face Med. 2023 Sep 8;19:42. doi: 10.1186/s13005-023-00385-y (PMC10486124; doi:10.1186/s13005-023-00385-y)
Supplement: Supplementary file 1 — Additional file 1: Supplement 1. [file 13005_2023_385_MOESM1_ESM.docx]

Supplement 1

Post-isometric muscle relaxation (PIR) treatments

During PIR treatments of the mandibular adductors, the patient was placed in a supine position with the head positioned neutrally. The series of treatments was carried out six times during one visit. The same physiotherapist performed the treatments under the same conditions.

While performing this technique, the therapist placed his thumbs on the patient's premolar and molar chewing surfaces and then passively retracted the mandible until the so-called functional barrier was reached. In this position, the patient performed an isometric contraction of the mandibular adductors, using about 20% of their maximum strength - the initiated contraction was balanced by the therapist's hands. After 10 s of isometric tension, the patient relaxed the muscles, and the therapist escorted the mandible to the new functional barrier. The described cycle was repeated thrice during a single treatment, starting from the previously obtained functional barrier [1].

3.2 Massage of the masseter muscle

During the massage treatments, the patient was placed in a supine position with the head positioned neutrally. The series of treatments was repeated twice during one visit. The same physiotherapist performed the treatments under the same conditions [1].

(a) intraoral massage

The massage was carried out on the right and then the left masseter muscle. The therapist positioned the thumb on the zenith of the patient's mouth and the index finger inside - 'pincer grip'. The massage was carried out by performing 10 vertical movements in a direction from the upper attachment to the lower attachment of the masseter muscle - band by band. Then, using the exact grip, 10 horizontal movements were carried out from the medial to the lateral side of the muscle in the same direction as above. The pressure applied by the therapist during therapy was approximately 0.5 kg during each maneuver.

(b) functional massage

With a pinch grip (as in 3.2a), the physiotherapist performed a vertical massage of the masseter muscle, during which the patient was asked to make 10 slow movements of opening and closing the mouth (to the limit of pain and/or discomfort). The pressure applied by the therapist during the therapy was approximately 0.5 kg during each maneuver.

3.3 Therapeutic exercises

Each patient was given a therapeutic exercise program, with instructions to perform them daily throughout the study [1].

a) Gerry's exercise

starting position: tongue positioned on the palate

movement: slow movements of opening and closing the mouth

number of repetitions: 6 times a day for 10 repetitions

b) Active exercises for lateral movements of the mandible

starting position: maxillary and mandibular teeth separated by about 5 mm

movement: the slow movement of the mandible to the left and back to the median line, then the direction of the mandible to the right and back to the median line

number of repetitions: 6 times a day for 10 repetitions

c) Side-to-side Exercise

Starting position: Place a pen or pencil in the mouth and hold it between your teeth.

Movement: Slowly move the jaw from one side to the other side. Repeat this exercise ten to fifteen times and three to five daily sets.

Number of repetitions: 6 times a day for 10 repetitions

(d) Protrusion and mouth opening

starting position: teeth separated

movement: a) lowering the jaw forward b) opening the mouth c) closing the mouth d) retracting the lower jaw

number of repetitions: 6 times a day for 10 movements

e) Cervical spine exercise (active flexion and extension movements of the spine).

Starting position: standing or sitting with head in neutral position (gaze straight ahead)

Movement: a) bend head and return to the initial position, b) straighten head and return to the initial position

number of repetitions: 6 times a day for 10 movements.

**References**

1 Chaitow L. Muscle Energy Techniques. 3rd ed. Wroclaw: Edra Urban & Partner; 2015.
